# Whole genome SNPs discovery in Nero Siciliano pig

**DOI:** 10.1590/1678-4685-GMB-2018-0169

**Published:** 2019-11-14

**Authors:** Enrico D’Alessandro, Domenico Giosa, Irene Sapienza, Letterio Giuffrè, Riccardo Aiese Cigliano, Orazio Romeo, Alessandro Zumbo

**Affiliations:** 1 Department of Veterinary Sciences, Division of Animal Production, University of Messina, Messina, Italy.; 2 Department of Chemical, Biological, Pharmaceutical and Environmental Sciences, University of Messina, Messina, Italy.; 3 Sequentia Biotech SL, Barcelona, Spain.; 4 Scientific Institute for Research, Hospitalization and Health Care (IRCCS) - Centro Neurolesi “Bonino-Pulejo”, Messina, Italy.

**Keywords:** Nero Siciliano pig, whole genome sequencing, variant calling, SNPs discovery, fitness genes

## Abstract

Autochthonous pig breeds represent an important genetic reserve to be utilized mainly for the production of typical products. To explore its genetic variability, here we present for the first time whole genome sequencing data and SNPs discovered in a male domestic Nero Siciliano pig compared to the last pig reference genome *Sus scrofa11.1*.A total of 346.8 million paired reads were generated by sequencing. After quality control, 99.03% of the reads were mapped to the reference genome, and over 11 million variants were detected.Additionally, we evaluated sequence diversity in 21 fitness-related loci selected based on their biological function and/or their proximity to relevant QTLs. We focused on genes that have been related to environmental adaptation and reproductive traits in previous studies regarding local breeds. A total of 6,747 variants were identified resulting in a rate of 1 variant every ~276 bases. Among these variants 1,132 were novel to the dbSNP151 database. This study represents a first step in the genetic characterization of Nero Siciliano pig and also provides a platform for future comparative studies between this and other swine breeds.

Preservation of genetic variability, used or potentially usable for food production, of non-food raw materials or related to social, cultural, and economic aspects, represents a challenge of fundamental importance on a planetary level. Although the difficulty of conserving biodiversity in species of zootechnical interest is not a recent concern, in recent years the need to preserve genetic variability within breeds has increased and is important in production systems (Kuec *et al.*, 2012). Animal genetic resources and their management systems are an integral part of ecosystems and productive landscapes in Italy, especially in Sicily. The role of livestock is more than ever, to provide sufficient food for humans that is protein-rich, safe and healthy, and with high nutritional and organoleptic values. Local pig breeds can be used for the production of raw materials particularly suitable for production of typical processed products. The higher economic value of typical productions compared to conventional commercial products and the growing consumer preference towards quality food could give support to plans for livestock biodiversity conservation (Herrero-Medrano *et al.*, 2013; Wilkinson *et al.*, 2013). In this respect, governments, institutional breeding organizations, private breeders and market demand play a crucial role in this endeavor toprotect and valuation of local breeds (Tapio *et al.*, 2006; Ollivier, 2009).

Although the interest in local pig breeds has increased significantly in recent years, only a few of them have been included in whole-genome sequencing projects (Esteve-Codina *et al.*, 2011, 2013; Herrero-Medrano *et al.*, 2014). The knowledge of the genetic background of these local breeds is very important as many of them have unique characteristics that could help address the challenges related to climate change, increase in world population and for food security and nutrition, as highlighted in the Domestic Animal Diversity Information System (DAD-IS) of FAO.

The Nero Siciliano pig is an autochthonous genetic type of the rural areas in Sicily (Italy). It lives in the woods of the Nebrodi and Madonie mountains and is reared in extensive and semi-extensive systems, making good use of pasture and other natural plant resources following the traditional practices used in this area. It is resistant to disease and with a great potential for adaptation to difficult environments, as it has a great ability for rooting and for procuring food. Its “Register of Native Breeds” was established in 2001 and now contains about 14.000 animals, of which ~5.000 are sows (ANAS - Italian Pig Breeders Association, 2018), from over 128 farms.The meat obtained from these pigs is sold at a higher price than that of commercial pigs, and in 2005 a request was made to allow labelling fresh Nero Siciliano meat with the Protected Denomination of Origin (D’Alessandro *et al.*, 2007). Black pigs are rustic, disease resistant animals, and live well in harsh conditions, but run a high risk of losing their original traits because of the lack of a real plan for genetic selection and setting up appropriate breeding systems and controls. The genetic variability of the Nero Siciliano pig has been assessed with the use of various genetic markers in several studies on molecular characterization of genetic structure and analysis of coat colour genes (*MC1R* and *KIT* gene) to evaluate their usefulness for breed traceability (Russo *et al.*, 2004; Fontanesi *et al.*, 2010; Guastella *et al.*, 2010).

All the procedures used in this research were in compliance with the European guidelines for the care and use of animals in research (Directive 2010/63/EU). A blood sample from a male of Nero Siciliano pig was used for DNA extraction. The individual was chosen for this study as one of the most representative boars of this breed, registered in the “Register of native breed” (ANAS; ID:163347). The leukocytes fraction recovered from the fresh whole blood sample was used for total genomic DNA (gDNA) extraction using the Wizard® Genomic DNA Purification Kit (Promega Corporation, Italy), following the manufacturer’s instructions. For DNA quantification a Qubit 2.0 Fluorometer was used with the Qubit dsDNA HS Assay Kit (Thermo Fisher, Italy). DNA quality was assessed by a Nanophotometer P-330 (Implen GmbH) and also by visual inspection after agarose gel electrophoresis (1% agarose in TAE 1X buffer). A PCR-Free library was prepared with TruSeq DNA kit (insert size 350 bp) using 1 μg of gDNA and following the protocol provided by Illumina. Paired-sequencing was carried out with a HiSeqX platform (Illumina).

The sequenced raw reads were checked using the FastQC program and cleaned with Trimmomatic v. 0.36 (Bolger *et al.*, 2014) to remove adapters and low-quality sequences (Phred score < 30). Good quality reads were mapped against the *Sus scrofa* reference genome (version 11.1; GenBank: GCA_000003025.6) with BWA (version 0.7.12-r1039) (Li and Durbin, 2009) and mapping quality was evaluated using Qualimap2 (Okonechnikov *et al.*, 2016). Single nucleotide polymorphisms (SNPs), short insertions and deletions (INDEL), and structural variants (SVs) analyses were performed using SUPERW and PINDEL pipelines (Ye *et al.*, 2009; Sanseverino *et al.*, 2015). The resulting variants were further filtered using the following parameters: QUAL (phred-scaled quality score of called variant) ≥ 30, DP (number of high-quality bases for called variant) ≥ 10, AD (allele depth) ≥ 10, removal of all called variants that showed the same genotype of the reference. Putative effects of SNPs were evaluated using SnpEff software v4_3m_core (Cingolani *et al.*, 2012). We further focused on 21 fitness-related gene sequences (Table 1) obtained using samtools (Li *et al.*, 2009) and bcftools (Li, 2011).

**Table 1:** List of 21 fitness related genes investigated in this study. The table shows the chromosome, gene symbol, gene function or putative gene association, starting and ending coordinates, reference.

Chr	Gene symbol	Gene function or putative association with QTLs	Start	End	Reference
1	ESR1	total newborn, newborn alive	14217032	14604906	Rothschild *et al*., 1996
1	VPS13A	maintenance of thermostatic status, blood circulation	230069339	230331343	Groenen, 2016
1	NR6A1	body size	265320597	265570941	Groenen, 2016
2	FSHB	total newborn, newborn alive	30395769	30399282	Zhao *et al*., 1998 [Bibr B51]
3	EIF2AK3	gene overlaps with QTLs for osteochondrosis score and feet and leg conformation	57423894	57506247	Laenoi *et al*., 2011
3	AZGP1	adaptation to environment	7867521	7874857	Beeckmann *et al*., 2003; Ma *et al*., 2009; Harmegnies *et al*., 2006
4	PLAG1	body size	75646585	75696718	Rubin *et al*., 2012
6	IL12RB2	immune related gene	145210251	145292399	Koch *et al*., 2012; Herrero-Medrano *et al.*, 2014
6	FUT1	resistance to disease	54077431	54080475	Meijerink *et al*., 1997, 2000; Bao *et al.*, 2012; Zhang *et al*., 2015; Fernández *et al*., 2017
8	GNRHR	Ovulation rate	65470206	65488900	Jiang *et al*., 2001
8	LCORL	body size	12806878	12969370	Rubin *et al*., 2012
9	AHR	litter size	86511866	86555950	Bosse *et al*., 2014
12	PPP1R1B	candidate genes affecting behaviour	22681244	22690978	Groenen, 2016
13	STAB1	immune related gene, defence against bacterial infection	34630448	34659371	Herrero-Medrano *et al*., 2014; Kzhyshkowska, 2010
13	GPR149	potential effect on fertility, prolificacy	94356371	94419917	Choi *et al*., 2015
13	CLDN1	potential effect on fertility	127714857	127730628	Choi *et al*., 2015
14	RBP4	total newborn, newborn alive	105037360	105044552	Rothschild *et al*., 2000
14	JMJD1C	potential effect on fertility	66640845	66966911	Choi *et al*., 2015
15	DCAF17	maintenance of thermostatic status, hair growth	77564629	77603913	Groenen *et al*., 2016
16	PRLR	involved in several reproductive traits, including litter size	20637568	20655881	Vincent et al., 1998; van Rens *et al*., 2003; Tomás *et al*., 2006
18	TAS2R40	adaptation to specific dietary repertoires and environment	7024418	7027197	Dong *et al*., 2009; Fischer *et al.,*2005,Ribani *et al*., 2017

Subsequently, the resulting high impact effects mutations were aligned and manually inspected with MEGA7 using the reference genomic and relative transcripts sequences retrieved from GenBank, in order to evaluate the putative functional role of the variants on the respective protein sequences. Variants called by SUPERW and PINDEL were compared with bedtools intersect and duplicates were removed from the PINDEL output. In order to detect novel SNPs, snpSift (Cingolani *et al.*, 2012) was utilized against dbSNP151 database (ftp.ncbi.nih.gov/snp/organisms/pig_9823/) and all resulting novel SNPs were manually examined and confirmed.

To explore the genetic resources of this breed, here we present for the first time the whole genome sequencing analysis of a male domestic Nero Siciliano pig, as well as a comparison with the most recent pig reference genome (Sscrofa 11.1) released by the International Swine Genome Sequencing Consortium and improved in annotation and assembly by Warr *et al.* (2015). In particular, we focused our attention on 21 genes that were selected according to their function and/or their association with specific traits (Table 1). These genes have been chosen because they affect phenotypes related to rusticity, adaptability to poor conditions of management and feeding, and great resistance to diseases, all these representing some of the most distinctive features of autochthonous breeds, especially the Nero Siciliano pig.

In this study, a total of 346.8 million raw paired-reads were produced by Illumina HiSeq X sequencing. After quality filtering and trimming, ~344.3 million (99.29%) high-quality reads were mapped to the *S. scrofa* reference genome, with a mean coverage of 39.5 X. A total of 11,253,945 genetic variants were detected by SUPERW in this study. Of these, ~82% were SNPs whereas ~12% and ~5% were short insertions and deletions respectively. Moreover, more than 58% of the detected SNPs (6,555,556 variants) were heterozygous, while the remaining 42% were found in alternative homozygosity state. The overall observed frequency was 1 variant every 222 bases, with a SNP mutation rate of 1/269 bp. However, we cannot confirm that all DNA mutations detected in this study segregate in the Nero Siciliano breed, as only one sample was considered.

SnpEff analysis showed that most of the variants recognized were located in non-coding regions of the genome, such as introns and intergenic regions (Figure 1a). Approximately 36% of the missense, 0.4% nonsense, and 63,6% silent mutations were observed, resulting in a missense/silent and Ts/Tv (transition/transversion) ratio of 0.5617 and 2.3956 respectively. However, the Ts/Tv ratio was similar to other pig genomes (Kang *et al.*, 2015), while the observed SNP mutation rate was slightly higher than that reported by Jungerius *et al.* (2005).

Among the structural variants identified by PINDEL, we observed a total of 808,486 insertions, 452,926 deletions, 196,971 replacements, 2,383 tandem duplications, and 1,029 inversions. Of these, 586,686 were heterozygous, whereas 875,109 were in alternative homozygosity.

Using the panel of fitness-related genes selected in this study, we identified a total of 6,747 SNPs and short INDELs (Figure 1b), that were classified according to Cingolani *et al.* (2012) in 7 “high”, 35 “moderate”, 54 “low impact” and 6,651 as modifiers (Table 2). This resulted in a mutation rate of 1 per ~276 bases; for further details see the supplementary material Tables S1, S2, and S3. Among the total variants identified, 1,132 were novel, consisting of 476 heterozygous and 656 in alternative homozygosity form.

**Table 2:** SNPs, short INDELs, and structural variants detected in the 21 fitness-related genes examined in this study.

Gene symbol	Length	Variants (SNPs and short INDEL) classified by impact				Total	%Variants/length	Structural variants
		High	Low	Moderate	Modifier			
ESR1	387875	0	14	0	2891	2905	0.749	102
VPS13A	262005	1	1	1	370	373	0.142	36
NR6A1	250345	0	0	1	9	10	0.004	13
FSHB	3514	0	0	0	0	0	0.000	0
EIF2AK3	82354	0	1	0	410	411	0.499	24
AZGP1	7337	2	4	7	66	79	1.077	2
PLAG1	50134	0	0	0	3	3	0.006	1
IL12RB2	82149	0	4	4	318	326	0.397	11
FUT1	3045	2	0	0	3	5	0.164	1
GNRHR	18695	0	0	0	25	25	0.134	3
LCORL	162493	1	4	1	422	428	0.263	30
AHR	44085	0	8	9	494	511	1.159	20
PPP1R1B	9735	0	0	0	13	13	0.134	2
STAB1	28924	0	1	3	9	13	0.045	1
GPR149	63547	0	9	4	370	383	0.603	10
CLDN1	15772	0	0	0	8	8	0.051	0
RBP4	7193	0	1	0	56	57	0.792	2
JMJD1C	326067	0	5	0	762	767	0.235	62
DCAF17	39285	0	1	2	178	181	0.461	10
PRLR	18314	1	1	3	240	245	1.338	15
TAS2R40	2780	0	0	0	4	4	0.144	0
TOTAL	1865648	7	54	35	6651	6747	0.362	345

**Figure 1:**
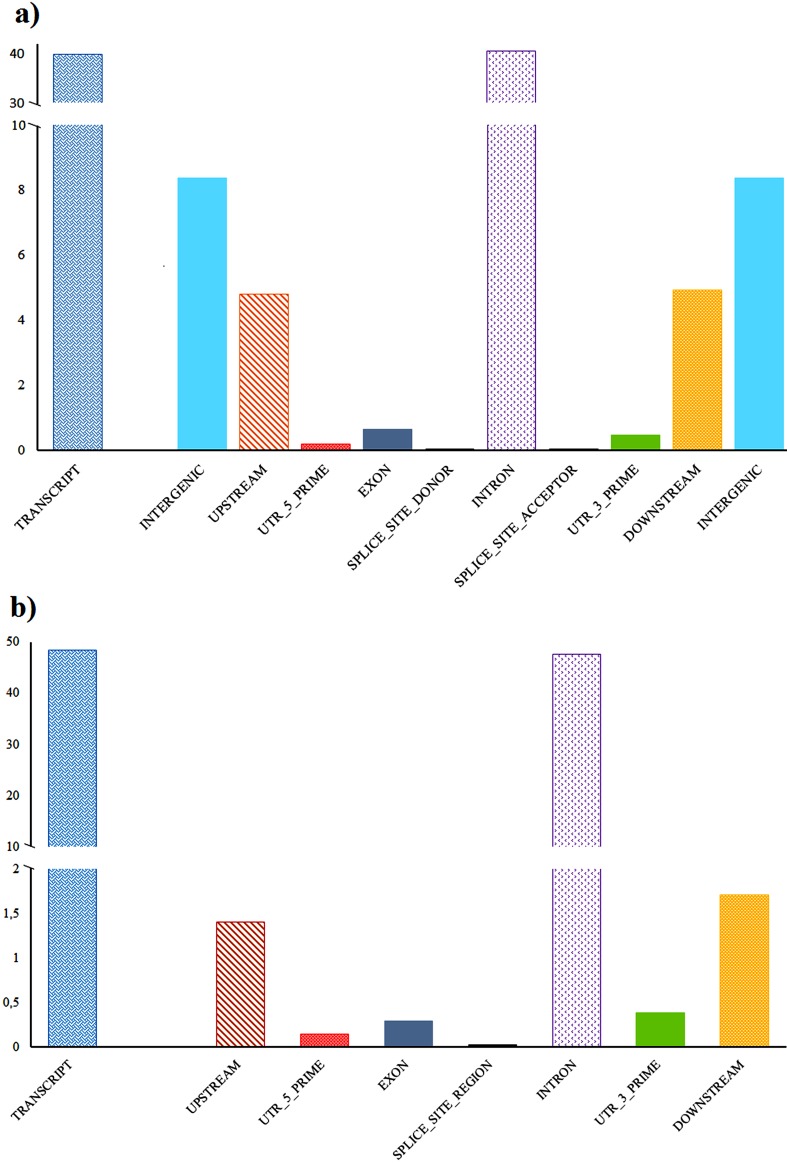
SNPs and short INDELs detected in this study in (a) whole genome and (b) in fitness related genes, and their location based on genomic annotation. Y-axis, represents the percentage of the variants.

The seven high impact mutations, all in the alternative homozygous state, affected five out of the 21 examined genes: *VPS13A* (Vacuolar protein sorting 13 homolog A*)*; *AZGP1 (*Alpha-2-glycoprotein 1, zinc-binding*); LCORL* (Ligand-dependent nuclear receptor corepressor-like protein*)*, *FUT1 (*Fucosyltransferases 1); *PRLR* (Prolactin Receptor). Such variants consisted in one SNP and six nucleotide insertions. Four of these latter were gain of function mutations and restored the reading frames of the *VPS13A, AZGP1, FUT1* and *PRLR* genes, as evidenced by comparative analysis with the reference genome and its transcripts. The remaining two insertions produced a premature stop codon and a lack stop codon in the *AZGP1* and *LCORL* genes respectively, whereas the unique SNP detected was a missense mutation (ACGàGCG; Thr^103^àAla^103^) affecting the *FUT1* gene. Five of these seven high impact mutations were novel to the dbSNP database (see Table S1).

Among the structural variations affecting the subset of the fitness-related genes we observed 101 replacements (RPL), 132 insertions, and 112 deletions. Of these, 203 were heterozygous and 142 were in the alternative homozygosity state. Figure 2 shows the gene-wide distribution of all detected mutations including the related sequencing coverage for all genes investigated.

**Figure 2:**
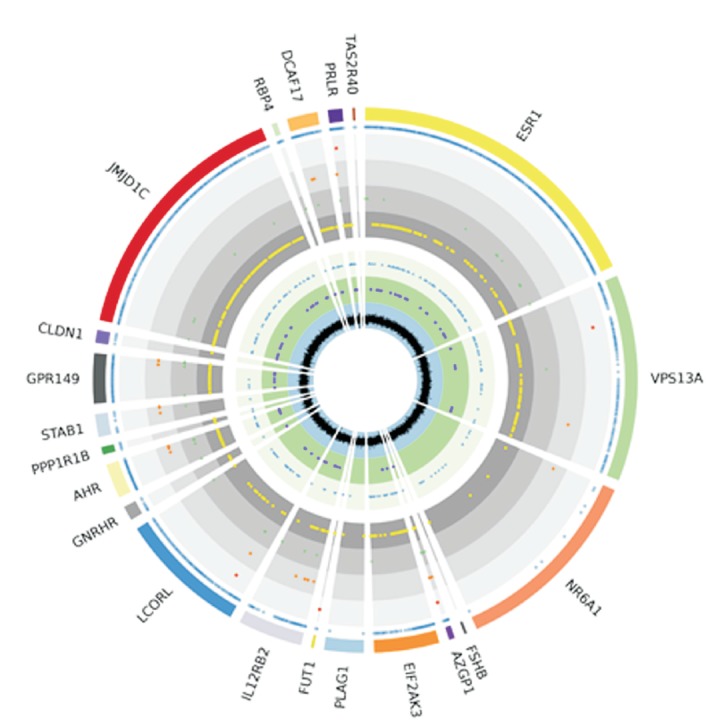
Variants detected in 21 fitness-related genes. From outside to inside, rings show: all SNPs and INDELs (blue circles), HIGH impact (red square), MODERATE impact (orange circles), LOW impact (green triangles), novel SNPs and short INDELs (yellow circles), SV INDEL (blue triangles), SV RPL (purple circles), reads coverage (black lines).

SNPs discovery analysis of the 21 fitness-related genes showed a coherent rate of mutation compared to the whole genome data. We focused on high impact mutations that may affect the gene product. The *VPS13A* gene plays a role in maintenance of thermostatic conditions during thermal stress and is involved in blood circulation (Groenen, 2016). We found a novel nucleotide insertion (G, genome position: 230125827) that causes a frameshift mutation restoring the *VPS13A* reading frame.

*AZGP1* is a putative candidate gene for adaptation to environment. A mutation in this gene, that overlaps QTLs for the number of vertebra (Beeckmann *et al.*, 2003; Harmegnies *et al.*, 2006), abdominal fat and ear shape and size (Ma *et al.*, 2009), was identified in Mangalica, Cinta Senese, and one European wild boar, but not in commercial pigs (Herrero-Medrano *et al.*, 2014). Furthermore, *AZGP1* is correlated with lipid mobilization and it is considered a candidate gene for body weight regulation and obesity in humans (Mracek *et al.*, 2010). We found two novel frameshift mutations in this gene, both nucleotide insertions (genome positions: 7874326 and 7874521 of the chromosome 3), which could affect its function, with possible effects on fat deposition. The *LCORL* gene overlaps a QTL involved in morphological modifications occurring during domestication events regarding elongation of the back and an increased number of vertebrae (Rubin *et al.*, 2012). This gene is considered a candidate gene for body size. A known *LCORL* frameshift mutation (rs791023757; genome position: 12829718) was detected in this study and resulted in a lacking stop codon. Unfortunately, no phenotypes have been associated so far with this mutation, as evidenced by lack of information in the dbSNP database.

We found two variants with high impact also in *FUT1* gene. This gene encodes a membrane protein involved in the synthesis of a precursor of blood group antigen. Previous studies showed that polymorphisms in this gene are associated with adhesion and colonization capacity of F18 fimbriated *Escherichia coli* to intestinal mucosa (Bao *et al.*, 2012; Zhang *et al.*, 2015). The toxins produced by this microrganism cause piglet post-weaning diarrhea (Luo *et al.*, 2010; Zhang *et al.*, 2015). We identified a missense mutation in position 54079560 of chromosome 6 (*FUT1* gene) that results in an amino acid change at position 103 (ThràAla) of the protein. This SNP, already recorded in the dbSNP database (rs335979375), was associated with *E. coli* F18-resistant or susceptible genotypes (Meijerink *et al.*, 1997, 2000). Tthe second identified variant was a G insertion (genome position: 54079637), but further studies will be needed to validate these findings and the role of these mutations in the Nero Siciliano breed.

The PRLR gene encodes a receptor for prolactin and is considered a strong candidate gene for various traits affecting directly (ovulation rate) or indirectly (ovarian weight, uterine length and number of teats) litter size and general reproductive performance in pigs (Vincent *et al.*, 1998; van Rens *et al.*, 2003; Tomás *et al.*, 2006). In the PRLR gene we detected a G insertion in position 20642378 (chromosome 16), but its contribution to the phenotypic variation remains to be elucidated.

The Nero Siciliano pig is not a well-characterised breed, and this study represents a first step in the genetic characterization of this animal, even if further research on the whole population reared in Sicily is needed to confirm the observed genetic variation and to integrate our data. In fact, all genetic changes detected in this study are only differences compared to the reference genome used and are therefore not indicative of the presence of mutated loci in the breed.

Since publication of the *Sus scrofa* reference genome (Warr *et al.*, 2015), several re-sequencing projects have been undertaken, but few have focused on local breeds. In this study we report, for the first time, the sequencing and variant calling analysis of a single boar of Nero Siciliano pig, with the aim of starting to acquire useful information on its genetic background that could be crucial to understand new genetic selection concepts for creating new sustainable pork chains based on local pig breeds. Therefore, the importance of preserving local breeds as a source of genomic diversity for further improvements of commercial pigs represents an added value in typical local productions. However, currently, in Italy the information regarding local pigs is strongly limited and therefore further sequencing studies will be essential for detecting the extent of genetic diversity occurring in Nero Siciliano pig.

The data sets supporting the results of this article are included within the article and its additional files. The raw reads used for *the genome-wide* analysis have been deposited in the NCBI Sequence Read Archive (SRA) under the following accession number: SRX3406507.

## Supplementary material

The following online material is available for this article:

Table S1 SNPs and short INDELs detected by SUPERW on fitness related genes and their classification into categories by SnpEff. Variants classified as high impact and related putative consequences on protein's functionalities.

Table S2 SNPs and short INDELs detected by SUPERW on fitness related genes and their classification into categories by SnpEff. Variants classified as moderate impact on protein function.

Table S3 SNPs and short INDELs detected by SUPERW on fitness related genes and their classification into categories by SnpEff. Variants classified as low impact on protein function.
