# Supplementary material for: Whole genome SNPs discovery in Nero Siciliano pig
Source: Genet Mol Biol. 2019 Nov 14;42(3):594–602. doi: 10.1590/1678-4685-GMB-2018-0169 (PMC6905442; doi:10.1590/1678-4685-GMB-2018-0169)
Supplement: Supplementary file 1 [file 1415-4757-GMB-42-3-2018-0169-20190905-suppl1.pdf]

Supplementary Material to "Whole genome SNPs discovery in Nero Siciliano pig"

Table S1 - SNPs and short INDELs detected by SUPERW on fitness related genes and their classification into categories by SnpEff. Variants classified as high impact and related putative consequences on protein's functionalities. SNP = single nucleotide polymorphism; short INDEL = short Insertions and deletion

Gene	Chromosome	Position	ID	Ref	Alt	Qual
VPS13A	CM000812.5	230125827	NEW	A	AG	214.458
AZGP1	CM000814.5	7874326	NEW	GCCC	GCCCC	214.458
AZGP1	CM000814.5	7874521	NEW	TCCCCC	TCCCCCC	161.457
FUT1	CM000817.5	54079560	rs335979375	T	C	221.999
FUT1	CM000817.5	54079637	NEW	AGG	AGGG	214.458
LCORL	CM000819.5	12829718	rs791023757	ATTTTTT	ATTTTTTT	124.457
PRLR	CM000827.5	20642378	NEW	TGG	TGGG	214.458
