# Supplementary material for: Whole genome SNPs discovery in Nero Siciliano pig
Source: Genet Mol Biol. 2019 Nov 14;42(3):594–602. doi: 10.1590/1678-4685-GMB-2018-0169 (PMC6905442; doi:10.1590/1678-4685-GMB-2018-0169)
Supplement: Table S2 [file 1415-4757-GMB-42-3-2018-0169-20190905-suppl2.pdf]

Supplementary Material to "Whole genome SNPs discovery in Nero Siciliano pig"

Table S2 - SNPs and short INDELs detected by SUPERW on fitness related genes and their classification into categories by SnpEff. Variants classified as moderate impact on protein function.

Gene	Chromosome	Position	Ref	Alt	Qual
VPS13A	CM000812.5	230276479	A	G	221.999
NR6A1	CM000812.5	265329130	T	C	225.009
AZGP1	CM000814.5	7870065	T	G	225.009
AZGP1	CM000814.5	7873624	A	G	225.009
AZGP1	CM000814.5	7874346	A	G	225.009
AZGP1	CM000814.5	7874418	G	A	225.009
AZGP1	CM000814.5	7874530	G	A	225.009
AZGP1	CM000814.5	7874548	G	C	225.009
AZGP1	CM000814.5	7874599	A	G	213.009
IL12RB2	CM000817.5	145214317	G	A	221.999
IL12RB2	CM000817.5	145224421	C	T	225.009
IL12RB2	CM000817.5	145229742	A	G	225.009
IL12RB2	CM000817.5	145245007	G	C	225.009
LCORL	CM000819.5	12830017	G	A	225.009
AHR	CM000820.5	86543037	A	T	225.009
AHR	CM000820.5	86543054	A	C	225.009
AHR	CM000820.5	86549936	A	C	225.009
AHR	CM000820.5	86550527	A	C	221.999
AHR	CM000820.5	86550544	GCTAC	GC	217.468
AHR	CM000820.5	86550673	TCAGCAAGACCTACA GCAAGACCTACAGCA	TCAGCAAGA CCTACAGCA	217.468
AHR	CM000820.5	86550830	G	T	225.009
AHR	CM000820.5	86551088	T	C	225.009
AHR	CM000820.5	86553513	A	C	198.009
STAB1	CM000824.5	34648222	T	C	225.009

Gene	Chromosome	Position	Ref	Alt	Qual
STAB1	CM000824.5	34656951	G	A	225.009
STAB1	CM000824.5	34658973	G	A	173.009
GPR149	CM000824.5	94411359	T	C	221.999
GPR149	CM000824.5	94411505	G	T	221.999
GPR149	CM000824.5	94417904	A	C	221.999
GPR149	CM000824.5	94418328	T	C	221.999
DCAF17	CM000826.5	77576067	A	G	225.009
DCAF17	CM000826.5	77579848	G	A	225.009
PRLR	CM000827.5	20637826	A	G	221.999
PRLR	CM000827.5	20638007	T	C	181.009
PRLR	CM000827.5	20638229	A	G	189.009
