# Supplementary material for: Whole genome SNPs discovery in Nero Siciliano pig
Source: Genet Mol Biol. 2019 Nov 14;42(3):594–602. doi: 10.1590/1678-4685-GMB-2018-0169 (PMC6905442; doi:10.1590/1678-4685-GMB-2018-0169)
Supplement: Table S3 [file 1415-4757-GMB-42-3-2018-0169-20190905-suppl3.pdf]

Supplementary Material to "Whole genome SNPs discovery in Nero Siciliano pig"

Table S3 - SNPs and short INDELs detected by SUPERW on fitness related genes and their classification into categories by SnpEff. Variants classified as low impact on protein function.

Gene	Chromosome	Position	Ref	Alt	Qual
ESR1	CM000812.5	14217032	T	C	225.009
ESR1	CM000812.5	14221367	T	C	173.009
ESR1	CM000812.5	14221457	A	G	225.009
ESR1	CM000812.5	14252100	C	T	225.009
ESR1	CM000812.5	14416426	A	G	225.009
ESR1	CM000812.5	14492048	T	C	221.999
ESR1	CM000812.5	14494565	A	G	225.009
ESR1	CM000812.5	14495006	G	A	217.009
ESR1	CM000812.5	14507705	G	A	209.009
ESR1	CM000812.5	14562995	T	A	225.009
ESR1	CM000812.5	14564951	T	A	225.009
ESR1	CM000812.5	14566227	A	G	225.009
ESR1	CM000812.5	14566274	G	A	225.009
ESR1	CM000812.5	14604479	C	T	225.009
VPS13A	CM000812.5	2,3E+08	G	A	221.999
AZGP1	CM000814.5	7867685	G	A	142.008
AZGP1	CM000814.5	7870110	G	A	225.009
AZGP1	CM000814.5	7874489	T	C	225.009
AZGP1	CM000814.5	7874855	A	T	225.009
EIF2AK3	CM000814.5	57505215	C	T	225.009
IL12RB2	CM000817.5	1,45E+08	T	C	225.009
IL12RB2	CM000817.5	1,45E+08	C	T	225.009
IL12RB2	CM000817.5	1,45E+08	T	C	221.999
IL12RB2	CM000817.5	1,45E+08	G	A	225.009
LCORL	CM000819.5	12829441	G	T	225.009
LCORL	CM000819.5	12830017	G	A	225.009
LCORL	CM000819.5	12830622	C	T	225.009
LCORL	CM000819.5	12968075	A	C	132.008
AHR	CM000820.5	86511891	C	T	111.008
AHR	CM000820.5	86542224	A	G	221.999
AHR	CM000820.5	86542269	G	A	225.009
AHR	CM000820.5	86549869	C	A	225.009
AHR	CM000820.5	86550001	A	C	225.009

Gene	Chromosome	Position	Ref	Alt	Qual
AHR	CM000820.5	86550097	C	T	225.009
AHR	CM000820.5	86550190	C	T	225.009
AHR	CM000820.5	86550235	A	C	218.009
STAB1	CM000824.5	34653589	C	T	225.009
GPR149	CM000824.5	94357750	A	G	221.999
GPR149	CM000824.5	94411372	G	T	221.999
GPR149	CM000824.5	94417810	C	G	221.999
GPR149	CM000824.5	94418113	A	G	221.999
GPR149	CM000824.5	94418125	A	C	221.999
GPR149	CM000824.5	94418490	A	G	221.999
GPR149	CM000824.5	94418551	A	G	221.999
GPR149	CM000824.5	94419514	G	A	221.999
GPR149	CM000824.5	94419904	G	A	221.999
JMJD1C	CM000825.5	66642024	A	G	221.999
JMJD1C	CM000825.5	66676717	A	G	221.999
JMJD1C	CM000825.5	66682302	A	G	221.999
JMJD1C	CM000825.5	66826098	T	C	221.999
JMJD1C	CM000825.5	66920177	G	A	221.999
RBP4	CM000825.5	1,05E+08	C	T	225.009
DCAF17	CM000826.5	77575138	C	A	225.009
PRLR	CM000827.5	20637918	T	A	225.009
